# High-Fat Diet Alters the Intestinal Microbiota in Streptozotocin-Induced Type 2 Diabetic Mice

**DOI:** 10.3390/microorganisms7060176

**Published:** 2019-06-16

**Authors:** Sheng Liu, Panpan Qin, Jing Wang

**Affiliations:** 1 BGI Education Center, University of Chinese Academy of Sciences, Shenzhen 518083, China; liusheng@genomics.cn (S.L.); qinpanpan@genomics.cn (P.Q.); 2 Institute of Quality Standard and Testing Technology for Agro-Products, Hubei Academy of Agricultural Sciences, Wuhan 430064, China

**Keywords:** high-fat diet, streptozotocin, type 2 diabetes, gut microbiota, 16S rRNA

## Abstract

Intestinal microbiota is closely associated with various metabolic diseases such as type 2 diabetes (T2D), and microbiota is definitely affected by diet. However, more work is required to gain detailed information about gut metagenome and their associated impact with diet in T2D patients. We used a streptozotocin-high-fat diet (HFD) to induce a T2D mouse model and investigated the effect of standard chow diet and HFD on the composition and function of gut microbiota. We found that a HFD could worsen the diabetes status compared with a standard diet. 16S rRNA gene sequencing revealed that a HFD caused a large disturbance to the microbial structure and was linked to an increased ratio of *Firmicutes* to *Bacteroidetes*. A HFD increased the bacteria of the *Ruminococcaceae* and *Erysipelotrichaceae* family and decreased the bacteria of *S24-7* and *Rikenellaceae*. Meanwhile, a HFD decreased the abundance of *Parabacteroides* *distasonis* and *Eubacterium* *dolichum*, both of which have previously been reported to alleviate obesity and metabolic dysfunctions. Moreover, PICRUSt-predicted KEGG pathways related to membrane transport, lipid metabolism, and xenobiotics biodegradation and metabolism were significantly elevated in HFD-fed T2D mice. Our results provide insights into dietary and nutritional approaches for improving host metabolism and ameliorating T2D.

## 1. Introduction

Diabetes is a metabolic disorder rapidly growing worldwide, and type 2 diabetes (T2D) accounts for more than 90% of patients with diabetes [1]. T2D is characterized by insulin resistance, pancreatic β-cell dysfunction, and increased fasting blood glucose [1,2]. The global transition towards diets high in processed foods, refined sugars, refined fats, oils, and meats is associated with increases in global incidences of chronic non-communicable diseases, especially T2D [3]. As we struggle to find solutions to T2D, a rapidly expanding area of research that is focused on the microbes that live within our digestive tract is offering fresh insights and potential avenues for intervention [4,5].

Several studies have indicated that obesity is associated with an increase in the phylum *Firmicutes* and a relatively lower abundance of the phylum *Bacteroidetes* [6,7]. Studies also showed that the gut microbiota promoted weight gain and hepatic steatosis in an FXR-dependent manner [8]. Moreover, Metformin is widely used in the treatment of T2D, and latest findings provide support for the notion that altered gut microbiota mediates some of metformin’s antidiabetic effects [9]. While genetics, physical environment, age, stress, and other factors can influence the dynamics of gut microbiota, diet can be the single most important driver of gut bacterial composition and function [10]. Dietary fibers could promote a select group of SCFA (short-chain fatty acid)-producing bacteria and induce changes in the entire gut microbe community that is associated with improved blood-glucose regulation in T2D [11].

To better study both the pathogenesis and potential therapeutic strategies, appropriate animal models of T2D are needed [12]. Since genetic models are costly and do not accurately model human T2D, the most commonly used animal model of T2D is the high-fat diet (HFD) fed rodent injected with streptozotocin (STZ) [13,14]. Gut microbiota in T2D have been studied for years [15,16], and HFD could determine the composition of the murine gut microbiome [17]. However, how standard diet or HFD modulate the intestinal microbiota in individuals with T2D deserves further research. Here, we used STZ and HFD to induce a mouse model of T2D and treated them with chow diet or HFD for four weeks. 16S ribosomal RNA (rRNA) gene sequencing was performed to assess the distinction of different diets on the composition of the gut microbiota in T2D mice. Our results will provide valuable insights into dietary and nutritional approaches for the prevention and management of T2D.

## 2. Materials and Methods

### 2.1. Animals and Experimental Design

**Figure 1:**
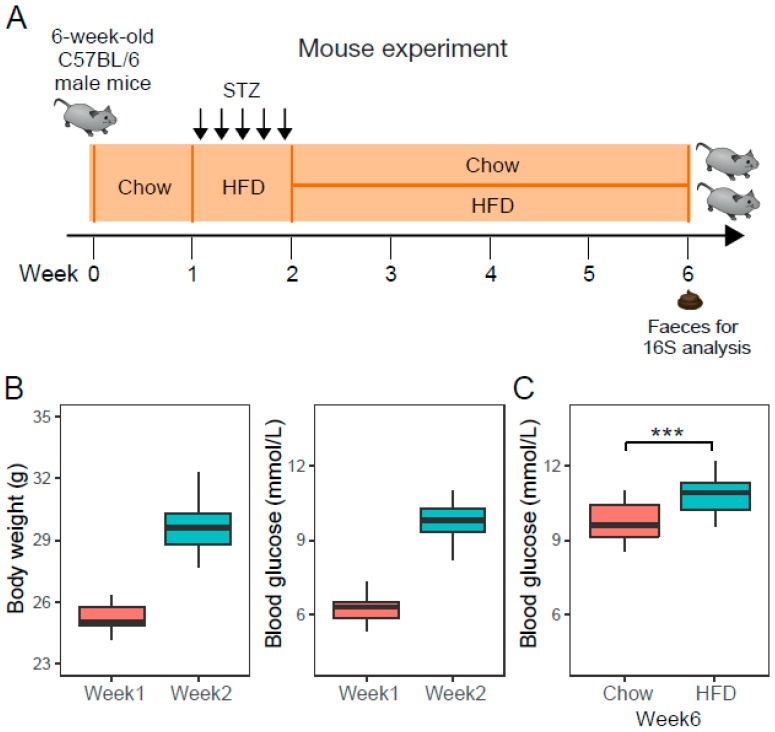
The experimental design and type 2 diabetes (T2D) mouse model. (**A**) Mouse experiment, 6-week-old C57BL/6 male mice (*n* = 50) were fed on a standard chow diet for one week, then injected with multiple low doses of streptozotocin (STZ) and fed on a high-fat diet (HFD) to obtain T2D mice. T2D mice received chow or HFD for four consecutive weeks. (**B**) Boxplot of body weight and fasting blood glucose in mice (*n* = 50) at the time point of week 1 and week 2. (**C**) Boxplot of fasting blood glucose in chow-fed T2D mice (Chow, *n* = 23) and HFD-fed T2D mice (HFD, *n* = 23) at week 6. *** denotes *p* < 0.001.

Animal experiments were conducted according to the guidelines approved by the ethic committee of Beijing Genomics Institute (ethics approval number: BGI-IRB 17185, and approval date: November 20, 2017). Fifty six-week-old C57BL/6 male mice were purchased from the Guangdong Experimental Animal Center and housed in a specific pathogen-free animal facility with a 12 h light-dark cycle and a temperature of 20 ± 2 °C. After a 1-week adaptation period, mice were fed HFD and intraperitoneally injected with low doses of STZ (40 mg/kg) for 5 days consecutively to achieve a diabetic state with limited morbidity and mortality (Figure 1A). At the end of the second week, fasting serum glucose and weight gain confirmed the development of T2D condition. T2D mice were randomly divided in two groups, receiving chow or HFD for four consecutive weeks, respectively. We assumed the change is gradual and becomes apparent after 4 weeks since hyperglycemia usually develops within 4 weeks of a high-fat diet [18]. The caloric composition of a standard chow diet is 20 kcal% protein, 70 kcal% carbohydrate and 10 kcal% fat, while the HFD contained 20 kcal% protein, 20 kcal% carbohydrate, and 60 kcal% fat.

### 2.2. Detection of Body Weight and Blood Glucose Level

The initial and final weights of the mouse models were measured by sensitive balance in order to investigate the effect of STZ-HFD on the body weight of mice. Fasting blood glucose (FBG) levels of experimental mice before and after STZ treatment were estimated using the Accu-Check Active digital glucometer.

### 2.3. Genomic DNA Extraction, 16S rRNA Gene Library Preparation and Sequencing

Feces samples derived from all mice were collected using a Qiagen stool kit at nine o’clock in the morning and immediately transferred to a −80 °C freezer. The total DNA was extracted using a previously published method [19]. The V4-V5 region of the bacterial 16S rRNA gene was amplified using specific PCR primers (515F 5′-GTGCCAGCMGCCGCGGTAA-3′, R926 5′-CCGTCAATTCMTTTRAGT-3′). The sequencing was performed on the Ion PGM™ platform [20] according to the protocols of the BGI-Shenzhen laboratory. The original 16S rRNA sequencing reads were deposited at the European Bioinformatics Institute (EBI) databases under the accession ID ERP113248.

### 2.4. Sequence Data Analysis

The 16S sequencing data were analyzed with pipeline based on Mothur v.1.33 [21] (The University of Michigan, Ann Arbor, USA). Alpha-diversity was assessed using ACE, Chao1, and Shannon indices. A UniFrac distance [22] metrics analysis was performed using the operational taxonomic units (OTUs) phylogenetic tree and abundance in each sample. We conducted principal coordinate analysis (PCoA) according to the matrix of the UniFrac distance of OTUs. Taxonomic classification of OTUs was assigned against the Greengenes database [23]. PICRUSt [24] was adopted to produce predicted KEGG Ortholog (KO) classifications with the 16S rRNA gene sequence data.

### 2.5. Statistical Analyses

Student’s t test was applied to assess whether any differences of body weight and blood glucose occurred in the two groups. Permutational multivariate analysis of variance (PERMANOVA) was used to assess the effects of different phenotypes on OTU profiles. OTUs or bacteria taxa that exhibited significant differences between two groups were identified using two-tailed Wilcoxon rank-sum tests and BH adjusted FDR < 0.05. Differentially enriched KEGG modules [25] were identified according to their reporter score [26] from the *Z*-scores of individual KOs. A reporter score of *Z* = 1.96 or *Z* = −1.96 was used as a detection threshold for significantly different modules.

## 3. Results and Discussion

### 3.1. STZ-Induced Type 2 Diabetic Mice Have Higher Body Weight Gain and Glucose Intolerance

The 50 mice were similar in regard to age, body weight, and glucose level before STZ treatment. Injections of multiple low doses of STZ, administered intraperitoneally on five consecutive days, induced higher body weight gain and impaired glucose tolerance (Table S1, Figure 1B). Mice with a fasting blood glucose (FBG) concentration exceeding 8.5 mmol/L were considered diabetic. 92% (46/50) of the mice were diabetic by the second week after STZ administration. Then, the 46 diabetic mice were randomly divided into two groups (Chow, *n* = 23; HFD, *n* = 23) to receive chow diet or HFD. After 4 weeks of different diets, T2D mice fed on the HFD significantly had a higher FBG (Figure 1C), indicating that a HFD could worsen diabetes status compared with a standard diet.

### 3.2. Overall Statistics and the Gut Microbiota Structure of the Feces Samples

We assessed gut microbiota by sequencing the 16S rRNA gene V4-V5 region. A total of 2,433,617 reads were obtained from 46 samples. Low-quality reads and reads shorter than 140 bp sequences were filtered, and then 729,915 reads were classified into OTUs with a 97% global similarity. We removed OTUs detected in less than 10% of the samples and finally obtained 1277 OTUs. The most abundant phyla included *Firmicutes* (814 OTUs) and *Bacteroidetes* (314 OTUs), contributing to 88.3% of all OTUs altogether. The dominant bacteria in all the mice were *Bacteroidetes* and *Firmicutes* phyla, which is consistent with human gut microbiota [27].

### 3.3. Structural and α-Diversity Changes of Intestinal Microbiota in STZ-Induced T2D Mice Fed on Chow and HFD

**Figure 2:**
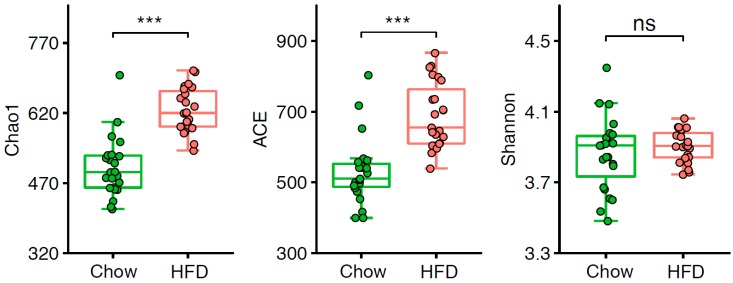
Alpha-diversity of the gut microbiota in two groups. Chao1, ACE, and Shannon indices were compared between chow-fed (*n* = 23) and HFD-fed (*n* = 23) T2D mice. *** *p* < 0.001, “ns” indicates no significance.

**Figure 3:**
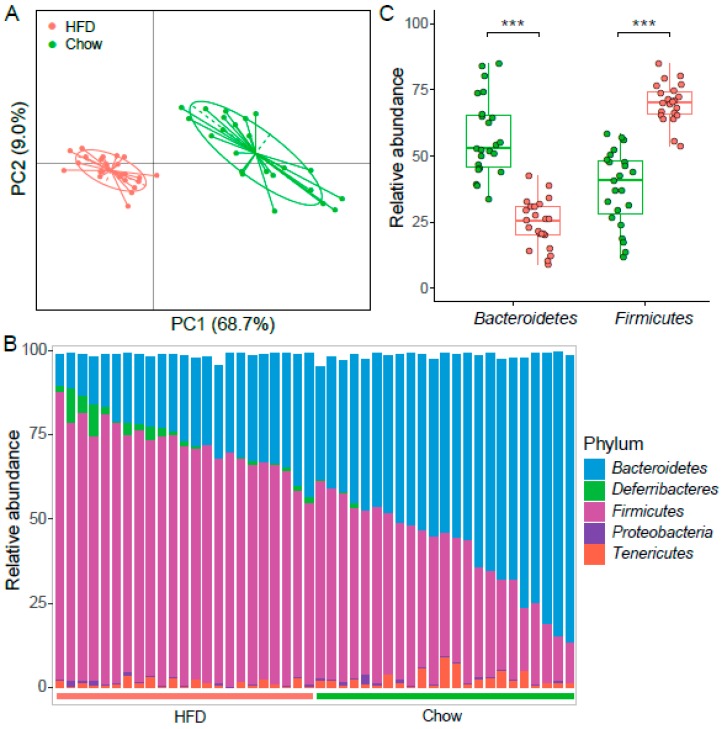
The structure of gut microbiota in two groups of mice. (**A**) PCoA based on weighted UniFrac distance of the OTUs abundance. The contributions of principal coordinate 1 (PC1) is on the X-axis and 2 (PC2) is on the Y-axis. (**B**) Bacterial taxonomic profiling at the phylum level among individuals is shown. Green bar represents chow samples, *n* = 23; red bar represents HFD samples, *n* = 23. (**C**) Boxplot of the relative abundance of *Bacteroidetes* and *Firmicutes* in samples from chow (*n* = 23) and HFD group (*n* = 23). *** denotes *p* < 0.001.

We examined the α-diversity of the intestinal microbiota in T2D mice and found Chao1 and ACE indices were significantly lower in the chow group than the HFD group (Figure 2, Table S2). However, there was no difference observed in the Shannon index, indicating that the community richness in mice on a HFD is considerably higher, but the diversity of the gut microbiota stable. We next sought to determine whether the structure of the microbiota could be modulated by HFD. PERMANOVA showed there was a significant difference of the abundance of OTUs between the chow group and the HFD group (Table S3). Moreover, PCoA of the weighted UniFrac distance based on OTU profiles showed substantial changes in the overall structure of the intestinal microbiota between these two groups (Figure 3A, Adonis *p* < 0.001).

### 3.4. HFD Altered the Intestinal Microbiota in STZ-Induced T2D Mice

To gain insight into the role of different diets on the intestinal microbiota, relative abundances of OTUs were compared between T2D mice fed on chow and a HFD. Among all 1277 OTUs, 699 OTUs (54.7%) were significantly different, and 58.3% (408/699) of them were enriched in the HFD group (BH adjusted FDR < 0.05). We next investigated the bacterial composition at taxonomic level. The most prevalent phyla were *Bacteroidetes* and *Firmicutes*, followed by *Proteobacteria*, *Tenericutes*, and *Deferribacteres* (Figure 3B). The abundance of *Firmicutes* increased significantly in the HFD group, while *Bacteroidetes* significantly increased in the chow group (FDR < 0.01, Figure 3C). Previous reports have shown that a high *Firmicutes*/*Bacteroidetes* (F/B) ratio can induce the development of obesity in mice and humans [28,29], and it is even significantly associated with BMI in a human population [30]. The F/B ratio increased in the HFD group, which is similar to results observed in obesity and chronic metabolic disease. Communities dominated by *Bacteroidetes* have been shown to have a protective effect against HFD induced glucose intolerance [31]. Moreover, the abundance of *Actinobacteria* and *Deferribacteres* were altered and became more abundant in the HFD group than the chow group (Table S4). Phylum *Deferribacteres* was reported to elevate in the gut microbiome of HFD-induced obesity [32]. *Deferribacteres* was dominated by *Deferribacteraceae* that had increased in dextran sodium sulfate (DSS)-induced colitis [33]. Taken together, HFD for T2D mice maintained the intestinal microbial characteristics of obesity and possible colitis, and on the contrary, the chow diet could weaken them.

The proportions of gut bacteria at the family level in HFD-fed mice were also very distinct from that in chow-fed mice (Figure 1). Wilcoxon rank-sum tests showed that the abundances of family *S24-7*, *Prevotellaceae*, and *Rikenellaceae* were enriched in the chow group, while *Ruminococcaceae*, *Erysipelotrichaceae*, *Mogibacteriaceae*, *Bacteroidaceae*, *Deferribacteraceae*, and *Lactobacillaceae* were enriched in the HFD group (FDR < 0.05, Table S5). Some members of the *S24-7* family can induce T-dependent responses [34] that are targeted by the IgA, suggesting that *S24-7* is involved in host-microbe interactions that impact on gut function and health [35]. Specifically, *Rikenellaceae* family members were suppressed in IBD patients relative to healthy controls [36]. We infer bacteria from *Rikenellaceae* might contribute to alleviating inflammatory disease, but a HFD probably exacerbates the inflammation in patients.

At the genus level, the abundances of *Oscillospira*, *Allobaculum*, *Blautia*, *Ruminococcus*, *Bacteroides*, *Mucispirillum*, *Dorea*, *Anaerovorax*, *Coprobacillus*, *Lactobacillus*, and *Tannerella* were significantly higher in the HFD group, while *Prevotella*, *Coprococcus*, *Turicibacter*, and *Rikenella* were significantly higher in the chow group (Figure 2, Table S6). In one human gut microbiota study, *Oscillospira* was enriched in lean subjects [37]. Notably, *Oscillospira* was strongly correlated with the fraction of secondary bile acids in the feces in both patients with gallstones and controls [38], indicating that it may contribute to the formation of secondary bile acids. Another report demonstrated that the improvement effects of acarbose on T2D were more remarkable in cluster B (rich in *Bacteroides*) than cluster *p* (rich in *Prevotella*), indicating that the decrease of *Bacteroides* abundance was beneficial in T2D treatment [39]. *Prevotella* strains are associated with plant-rich diets but are also linked with chronic inflammatory conditions [40]. Previous research reported *Rikenella* was less abundant in mice with HFD-induced steatohepatitis [41]. Therefore, chow diet contributed to increasing *Rikenella*, which may have positive effects on alleviating obesity and associated diseases. In addition, the representation of *Blautia* in prediabetes patients was higher than in patients with normal glucose tolerance and even higher in T2D [42], so high *Blautia* abundance together with a high-fat diet was associated with T2D.

**Figure 4:**
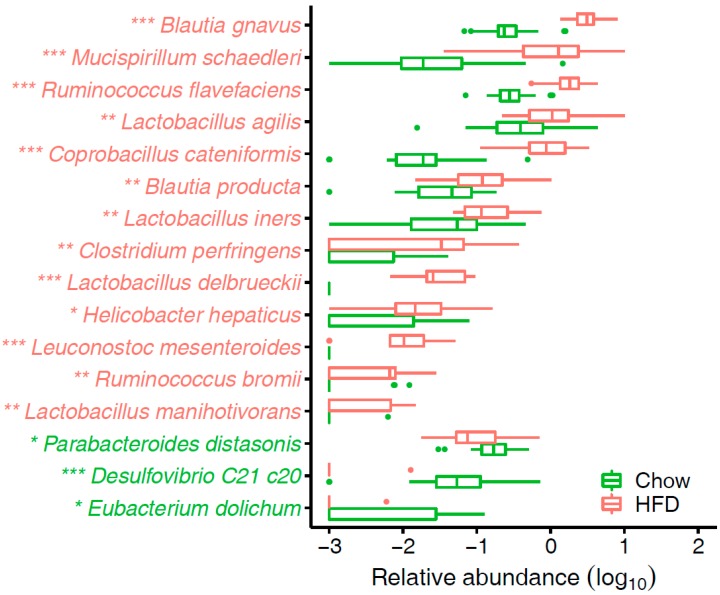
Species that significantly differ in abundance between chow group and HFD group. * FDR < 0.05, ** FDR < 0.01, *** FDR < 0.001, FDR-controlled Wilcoxon rank-sum test. The species names are colored according to significant differences: green, enriched in chow group; red, enriched in HFD group.

Detailed analysis indicated that the enriched species in T2D mice fed on a HFD were *Blautia gnavus*, *Blautia product*, *Coprobacillus cateniformis*, *Mucispirillum schaedleri*, *Ruminococcus flavefaciens*, *Lactobacillus agilis*, *Lactobacillus iners*, and *Clostridium perfringens* (Figure 4, Table S7). *B. product* was found at considerably higher levels in rats fed a HFD than control chow, and it was involved in acetate or propionate production [43]. In line with the result, Zhang et al. [44] reported significantly increased levels of acetic acid and propionic acid in rats fed a HFD. *C. catenaformis* is a new species that has been isolated from human feces in Japan [45], but we know very little about its function. Researchers found that *M. schaedleri* has specialized systems to handle oxidative stress during inflammation, and additionally, it can modify the mucosal gene expression of its host [46]. Another study showed that nopal treatments reduced the obesity-related biochemical abnormalities and increased the adundance of *Ruminococcus flavefaciens*, *Bacteroides fragilis*, and *Akkermansia muciniphila* [47]. *A. muciniphila* are beneficial microbes that have been inversely associated with body fat mass and glucose intolerance [48], but the function of *R. flavefaciens* warrants further study. *L. iners*-dominant cervical microflora had a significant association with obesity [49]. Biochemical and functional assays suggest that *L. iners* bacteria contain features of probiotic lactobacilli as well as of vaginal pathogens [50]. Different *Lactobacillus* species are associated with different effects on weight change that are host-specific [51]. Further studies are needed to clarify the role of *Lactobacillus* species in human energy harvest and weight regulation. *C. perfringens* is one of the most common causes of food poisoning in the United States [52] and is associated with several exotoxin-mediated clinical diseases.

By contrast, *Parabacteroides distasonis*, *Desulfovibrio C21 c20*, and *Eubacterium dolichum* were enriched in chow-fed T2D mice (Figure 4, Table S7). A recent study indicated *p. distasonis* modulates host metabolism and alleviates obesity and metabolic dysfunctions via production of succinate and secondary bile acids [53]. Genome sequencing and analysis of *E. dolichum* showed it encodes a beta-fructosidase capable of degrading fructose-containing carbohydrates such as sucrose, genes for the metabolism of PTS (phosphotransferase system)-imported sugars to lactate, butyrate, and acetate [54]. And these short-chain fatty acid (SCFA) products could protect against diet-induced obesity [55]. Collectively, T2D mice take proper diets could bring more advantages to metabolism over HFD.

### 3.5. Functional Alterations in the Gut Microbiome of T2D Fed on Chow Diet and HFD

**Figure 5:**
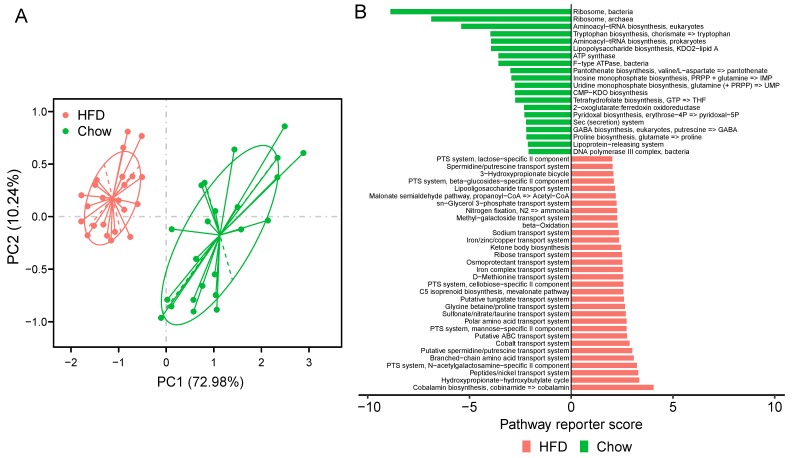
KOs and modules in samples of chow and HFD group. (**A**) PCA of the KO profile between the two groups. Chow samples, *n* = 23 (green); HFD samples, *n* = 23 (red). (**B**) Differentially modules between chow and HFD. Reporter score ≤ −1.96, blue color; reporter score ≥ 1.96, red color.

To better understand the functional capabilities of the microbiota, we adopted PICRUSt to predict the functional composition of a metagenome. PCA showed that the KO profile of the gut microbiota in chow-fed T2D mice diverged from that in HFD-fed T2D mice. The first principal component (PC1) explains 72.98% of the variation and separates the mice in the chow group and HFD group (Figure 5A). Moreover, PERMANOVA analysis indicated that the KOs exhibited a significant distinction between the two groups of mice (adonis test, *p* < 0.001). These data suggest that HFD could result in a notable disturbance in the functions of gut microbiota of T2D mice. When considering KEGG pathways, 119 pathways were altered between the chow group and the HFD group, and 83 were over-represented in the HFD group (FDR < 0.05, Table S8). Pathways related to membrane transport and lipid metabolism were significantly elevated in HFD-fed mice, including ABC transporters, phosphotransferase system (PTS), fatty acid biosynthesis, and fatty acid metabolism. The HFD also enriched the pathway of xenobiotics biodegradation and metabolism. Interestingly, previous studies indicated that T2D-enriched markers were typically involved in the KEGG categories of membrane transport and xenobiotics degradation and metabolism [15].

Furthermore, at the module level, 52 modules varied significantly between the two groups of mice (Figure 5B, Table S9). In T2D mice fed a HFD, we observed the striking enrichment of many modules of the PTS, which are responsible for transporting glucose through outer and inner membranes and catalyzing the uptake of carbohydrates. The increased relative abundance of these pathways indicate that the HFD for a T2D status may stimulate accelerated bacterial usage of glucose as energy. Modules involved in the iron/zinc/copper transport system were also more abundant in HFD-fed mice, while modules of Ribosome and Lipopolysaccharide biosynthesis were enriched in chow-fed T2D mice. Iron, zinc, and copper uptake systems significantly contribute to the virulence of many pathogenic bacteria [56], so a HFD may trigger the gut microbiota to produce virulence factors. These findings suggest that functionally, the microbiota could be perturbed by a HFD in T2D mice.

## 4. Conclusions

We comprehensively examined the structural and functional analysis of the intestinal microbiota in STZ-induced T2D mice. The results of alpha-diversity analysis demonstrated that the community richness in T2D mice fed on a HFD is considerably higher than in those fed on chow. However, in terms of overall diversity, there was no significant change. PERMANOVA analysis showed that a HFD not only significantly changed the components of the gut microbiota in T2D mice, but also changed the functional pathways compared to those fed a chow diet. HFD increased the F/B ratio in the gut and decreased the bacteria from family *S24-7* and *Rikenellaceae.* Furthermore, the abundance of *Parabacteroides distasonis* and *Eubacterium dolichum* were down-regulated in the HFD group.

Functional analysis of inferred metagenomes revealed pathways related to membrane transport, lipid metabolism, and xenobiotics biodegradation and metabolism were significantly elevated in HFD-fed T2D mice. We also observed that a HFD enriched many modules of the PTS and iron/zinc/copper transport system, which may exacerbate T2D. The study suggests that compared to a HFD, a standard diet appears to be an effective strategy for improving host metabolism and ameliorating diabetes-mediated disorders in T2D individuals.

## Supplementary Materials

The following are available online at https://www.mdpi.com/2076-2607/7/6/176/s1, Table S1: The body weight and fasting blood glucose (FBG) in mice, and FBG concentration exceeding 8.5 mmol/L were considered diabetic, Table S2: The alpha-diversity indices of the fecal samples in mice (Chow, *n* = 23; HFD, *n* = 23), Table S3: PERMANOVA showed there were was a significant difference in the abundance of OTUs between chow group and HFD group, Table S4: Phyla that differ in abundance between chow group and HFD group, Table S5: Families that significantly differ in abundance between chow group and HFD group (FDR < 0.05), Table S6: Genera that significantly differ in abundance between chow group and HFD group (FDR < 0.05), Table S7: Species that significantly differ in abundance between chow group and HFD group (FDR < 0.05), Table S8: PICRUSt predicted KEGG pathways that significantly differ in relative abundance between chow group and HFD group (FDR < 0.05), Table S9: The significantly different distributed KEGG modules between chow group and HFD group (Zscore < −1.96, enriched in chow group; Zscore > 1.96, enriched in HFD group).
